# A Successful Treatment of COVID-Induced Acute Idiopathic Pancreatitis with an RNA-Polymerase Inhibitor Agent

**DOI:** 10.7759/cureus.51992

**Published:** 2024-01-10

**Authors:** Ana P Urena Neme, An Tran, Michael Victoria Guerrero, Gabriella Roa Gomez, Miguel A Rodriguez Guerra

**Affiliations:** 1 Medicine, Medicina Cardiovascular Asociada, Santo Domingo, DOM; 2 Medicine, Montefiore Medical Center, New York, USA; 3 Medicine, Instituto Tecnológico de Santo Domingo, Santo Domingo, DOM; 4 Pulmonary and Critical Care Medicine, Albert Einstein College of Medicine, Bronx, USA; 5 Pulmonary and Critical Care Medicine, Montefiore Medical Center, Wakefield Campus, Bronx, USA; 6 Medicine, Montefiore Medical Center, Albert Einstein College of Medicine, Bronx, USA

**Keywords:** nucleotide analog, rna polymerase inhibitor, acute sars-cov-2, remdesivir, covid pancreatitis

## Abstract

Acute idiopathic pancreatitis (AIP) has been rarely linked to SARS-CoV-2 and its mechanism is not completely understood. As a result, its management, due to the heterogeneity of the literature, may pose a challenge. This case report describes a 59-year-old female who presented to the emergency department with severe epigastric pain, fever, and a positive SARS-CoV-2 polymerase chain reaction (PCR) test. Imaging confirmed acute interstitial pancreatitis, which was successfully managed using the viral RNA polymerase inhibitor, remdesivir. Pancreatitis-associated complications, such as sepsis and shock, are recognized as significant factors contributing to extended hospitalization and increased mortality rates. The management of autoimmune pancreatitis poses a challenge due to the diverse existing literature, resulting in a lack of standardized approaches. Although the impact on inpatient mortality of remdesivir remains uncertain, early administration of RNA polymerase inhibitors could alleviate complications and positively impact the duration of hospitalization. Further research is important to create optimal management strategies for complications related to COVID-19-related pancreatitis.

## Introduction

The most common causes of acute pancreatitis (AP) are gallstones and alcoholism [1]. However, infectious agents such as viruses, bacteria, and parasites are known to trigger AP [2]. SARS-CoV-2 infection is not commonly associated with gastrointestinal symptoms [3]. Emerging data suggests that the pancreas may be a target organ. Its pathogenesis is still unclear, but the hypothesis is a possible inflammation of the pancreatic glands leading to acute idiopathic pancreatitis (AIP) [4,5]. This case involves a patient with AIP secondary to SARS-CoV-2 infection successfully treated with a viral RNA polymerase inhibitor agent.

## Case presentation

**Figure 1:**
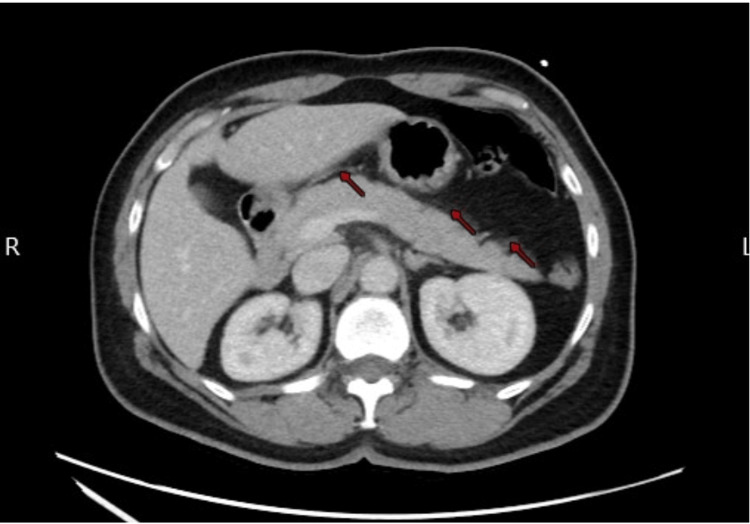
CT evidenced the pancreatic fatty stranding.

A 59-year-old Hispanic female with a past medical history of gastritis, gastroesophageal reflux disease (GERD), and chronic back pain presented to the emergency department (ED) complaining of severe epigastric pain and subjective fever at home for four hours, associated with fatigue and nasal congestion. She denied any family history or prior symptoms. During the physical examination, she appeared distressed due to epigastric pain, with a blood pressure of 143/83 mmHg, a heart rate of 116 bpm, and a temperature of 101.7°F. Laboratory evaluation was notable for elevated lipase (658 U/L), C-reactive protein (15.7 mg/L), and positive SARS-CoV-2 polymerase chain reaction (PCR) test. Upon admission, an abdominal US was negative for gallstones and the abdominal and pelvic CT showed pancreatic fatty stranding indicative of AIP without gallstones or biliary duct dilation (Figure 1).

The patient received ringer lactate at 100 cc/hr for 24 hours and symptomatic treatment. Infectious disease was consulted due to AP in the setting of SARS-CoV-2 and recommended a five-day course of therapy with remdesivir. The patient responded well to the treatment, and her lipase started to downtrend (Table 1).

**Table 1:** Lipase trending

	Day 1	Day 2	Day 3	Day 4	Reference
Lipase	658	474	236	41	8-78 U/L

The patient only received fluid IV for the first day and was encouraged to increase her oral intake. Later, on the fifth day, she was discharged after completing her remdesivir course, with a follow-up scheduled at the discharge clinic with gastroenterology.

## Discussion

AIP secondary to COVID-19 infection is uncommon [6]. The available literature is heterogeneous; Chaudhry et al. reported a cohort from the national inpatient sample database and exposed that less than 1% of patients with this infection have presented AIP. It is more common in males and Caucasian patients. They also reported that these patients had a higher incidence of fungal infections and the mean length of stay (LOS) was 10.53 days [7]. However, Pandanaboyana et al. reported an international multicenter cohort study revealing a prevalence of 8.3% [8]. Yang et al. reported a study that included patients from China, Europe, and the US, exposing a prevalence of 3.1% [9]. In New York hospitals, Inamdar et al. found an AIP prevalence of 0.27% [10].

Pancreatitis is known to be associated with a higher incidence of sepsis, shock, longer LOS, and mortality [11,12]. The development of sepsis and shock can be seen as a result of marked arterial vasoconstriction leading to volume deficit and subsequently shock. Wilson et al. described another mechanism in which angiopoietin-2 levels cause endothelial cell dysfunction and vascular leak syndrome, leading to sepsis and shock [13].

The management of AP depends on its etiology (ursodeoxycholic acid, cholecystectomy, endoscopic sphincterotomy, pancreatic enzyme, antioxidant, somatostatin, or octreotide). However, in cases of unclear etiology, due to the heterogeneity of the literature, the management or recommendations may pose a challenge [14-17].

Recent data suggests that COVID-19 significantly worsens the outcomes for individuals with AP. In the analysis made by Chaudhry, patients with AP had statistically significantly higher mortality (P=0.02), this was also seen in a systematic review by Mutneja in which patients that had pancreatitis secondary to COVID-19 had a greater mortality rate (OR 4.10, 95% CI 2.03-8.29) and acute kidney injury was also 78% higher in patients with pancreatitis and COVID-19 [7].

Moreover, individuals experiencing both conditions showed higher rates of severe pancreatitis (OR 3.51, 95% CI 1.19-10.32), necrotizing pancreatitis (OR 1.84, 95% CI 1.19-2.85), and an extended hospital stay (OR 2.88, 95% CI 1.50-5.52) compared to those without COVID-19. Additionally, patients with COVID-19 were more likely to have AP with an unknown or idiopathic cause (OR 4.02, 95% CI 1.32-12.29) compared to their counterparts without COVID-19. These findings emphasize the worsened clinical outcomes associated with the simultaneous presence of COVID-19 in individuals diagnosed with AP.

SARS-CoV-2 management includes remdesivir, a nucleotide analog with an antiviral activity agent that inhibits the RNA-polymerase, affecting viral replication and leading to an early termination of the viral cycle. The therapy is initiated with 200 mg of remdesivir first, followed by 100 mg daily; its course may vary up to 10 days [18]. This therapy has positively impacted the LOS but the inpatient mortality benefit remains uncertain [19]. This therapy has positively impacted the LOS but the inpatient mortality benefit is uncertain [20].

In summary, pancreatic injury in COVID-19 is uncommon, and its consequences may potentially lead to death. The LOS in a COVID patient with pancreatitis is expected to be prolonged. Our report is a unique case of an uncomplicated AIP induced by SARS-CoV-2 that was successfully treated with RNA-polymerase inhibitor resulting in a within five days discharge.

## Conclusions

This case highlights an uncommon instance of AIP secondary to SARS-CoV-2 infection, successfully managed with a viral RNA polymerase inhibitor, remdesivir. Although the impact of this treatment on inpatient mortality is uncertain, this may suggest that an early approach with this agent can avoid potential complications and may positively impact the LOS in these patients. Further research is needed to clarify the optimal management strategies for COVID-19-related pancreatitis.
